# Improving experiences of transgender patients in inpatient services through a ward based staff training program

**DOI:** 10.1192/bjo.2021.594

**Published:** 2021-06-18

**Authors:** Noah Stanton, Phoebe Lyons, Dr Amy Barsby

**Affiliations:** Lancashire Care NHS Foundation Trust

## Abstract

**Aims:**

The aim was to improve the experience of transgender patients in a general adult inpatient setting, through delivering practical 'bitesize' ward-based staff training. This training was to improve awareness of issues faced by transgender patients, knowledge around gender dysphoria, and increase confidence in discussing these issues appropiately with patients.

**Method:**

Staff from a range of disciplines attended sessions held on the ward in small groups; these bite size sessions were delivered in under 20 minutes making them easy to fit around clinical commitments.

**Result:**

All attendants rated increased confidence in their skills and ability to support transgender patients.

**Conclusion:**

Improved staff training specifically focussing on transgender patients can contribute towards improved care for this patient group; this should form part of a wider strategy including clear operational policy and supportive environments.

